# Perceptions of nurses on occupational health hazards and safety practices in Ditsobotla public hospitals in North West province

**DOI:** 10.4102/curationis.v45i1.2220

**Published:** 2022-07-15

**Authors:** Takalani Denge, Mahlasela Rakhudu

**Affiliations:** 1 Department of Nursing Science, Faculty of Health Science, North-West University, Potchefstroom, South Africa

**Keywords:** hazards, health settings, nurses, occupational hazards, occupational health perceptions, public hospitals, risk and safety

## Abstract

**Background:**

Nurses are the backbone of the healthcare system. During the fulfilment of their duties and responsibilities, they experience various types of work-related risks, which harmfully affect their health and nursing quality.

**Objectives:**

This study aimed to explore and describe perceptions of nurses on occupational health hazards and safety practices in Ditsobotla public hospitals of North West province.

**Method:**

An exploratory, descriptive, qualitative research design was undertaken in this study. An explorative design allowed the researcher to identify key issues regarding nurses’ perceptions on occupational health hazards and safety practices using Donabedian structure, process and outcome. A total of 15 nurses of different categories participated in the study that formed four focus group discussions. Semi-structured focus group discussions of three to four participants were conducted until data saturation is obtained and at the point where no new themes from participants’ perceptions emerged. Creswell and Clark framework of data analysis was used to analyse data.

**Results:**

Three major categories emerged during data analysis: nurse’s perception on occupational health hazards in the health settings, Donabedian framework on assessing the quality of care in relation to occupational health hazards and occupational health and safety (OHS) practices. Eight themes were identified.

**Conclusion:**

Nurses’ perceived different occupational hazards that affect their normal duties and responsibility in the workplace. Future interventions such as training and education on OHS policy should be adopted to promote health and well-being of the staff.

## Background

Nursing profession deals with the health and most importantly, life of the patients and society at large although it demands a great deal of commitment. Nurses are confronted with many occupational hazards during the course of discharging their duties (Chiou et al. 2013:1377) and continue to report high levels of job-related injury and illness (Irtyah 2014:59). Working environment and nurse’s responsibilities put them in the front line of numerous occupational health hazards, leading to acute or health problems. Walton and Rogers (2017:544) identified biological, physical, chemical and psychosocial hazards as common occupational health hazards in the health setting.

The World Health Organization (WHO 2012:2) estimated the global burden of diseases from occupational exposure to be 40% worldwide. Each year, there are thousands of non-fatal occupational injuries, which are estimated to affect 374 million workers every year (Hämäläinen, Takala & Kait 2017:20). It is estimated that yearly, over two million people worldwide die of occupational injuries and work-related diseases. The magnitude of these hazards necessitate the need to explore perceptions of nurses on occupational health hazards and safety measures in North West province.

According to International Labour Organization (2016:12), the most common accidents prevailing in health settings include blood spillage, falling, needle pricks, infections and psychosocial hazards. In Nigeria, Ghahremani et al. (2018:56) introduced four main reasons regarding hospital incidents. These incidents included the lack of access to appropriate protective equipment, behaviour of staff, inadequacy of tools and excessive tendency towards high-speed performance. Hundreds of millions of people throughout the world are working under circumstances that foster ill health and are unsafe.

Nurses are exposed to physical hazards such as needle stick injuries in their workplace leading to the risk of infections (Nui 2016:10). This is supported by Nophale (2009:30), who conducted a study on needle stick injuries amongst healthcare workers (HCWs) in regional hospitals in the Free State province of South Africa. The results revealed that 90% of professional nurses and 55 (6%) of doctors sustained needle stick injuries. Owie and Apanga (2016:596) found that the increase in occupational health hazards in developing countries is mostly blamed on healthcare professionals who do not practice universal safety measures such as hand washing, wearing gloves and using personal protective equipment. This unsafe practice increases the risk of injuries and transmission of infections to HCWs.

Moreover, nursing students are also exposed to occupational hazards and injuries in the course of their clinical training activities because of inexperienced, unskilled and lack of professional knowledge of protective measures than senior nurses (Eljedi 2015:34). Exposure to infectious materials can be minimised by adherence to standard precautions, which are designed to reduce the risk of acquiring occupational infection in the healthcare settings.

The occupational health nurse plays a multifaceted role in influencing, improving or maintaining a worker’s daily workplace health using prevention, protection and health intervention and creating productive healthy workers in a healthy workplace (Tudor et al. 2014:18). The *Occupational Health and Safety Act* (Act 85 of 1993) requires medical surveillance to be conducted and to manage the risks identified (Tudor et al. 2014:18). Based on data available on health hazards and occupational health and safety on health professions, there is a dearth of literature in North West province because there is no study conducted on occupational health hazards and safety practices.

## Statement of research problem

The researcher has been working in the research area for the past two years as a professional nurse and observed with concern the eminent occupational risks affection provision of healthcare. Nursing profession deals with health and most importantly life of the people in the society, and hence it demands a great deal of commitment. Because of the nature of their work, nurses are at risk of so many occupational hazards. These hazards include cuts, bruises, accidental needle stick injuries and infections caused by bacteria and viruses such as human immunodeficiency virus (HIV) and coronavirus disease 19 (COVID-19), which can be transmitted through contact with infected patients or contaminated objects, body secretions, tissues or fluids (Owie & Apanga 2016:596). Although nurses at Ditsobotla public hospitals face such hazards, this area is largely under-researched. According to International Labour Organization (2016:12), employers in rural areas are faced with a loss of skilled staff, migration and early retirements from nursing professionals as a result of exposure to health hazards in the workplace. Therefore, it is important to explore perceptions of nurses on occupational health hazards and safety practices.

Regardless of legislation in South Africa to address the challenges of occupational health hazards, much is not known about the extent to which nurses at Ditsobotla public hospitals are complying with written protocols designed to prevent and mitigate health hazards at the workplace. Literature demonstrated an increase in the number of cases of occupational hazards in health settings and a major gap in the safety practices (Tudor et al. 2014:18). However, the result of this study will generate more insight into gap that exists in meeting the occupational health and safety. Findings from this study would be useful for other researchers following the publication to fill the existing gap.

## Conceptual framework

Donabedian theory provides a framework for examining health services and evaluating the quality of healthcare. The author identified a three-component approach for evaluating the quality of care including structure, process and outcomes. Donabedian (1988:1743) believed that structure measures have an effect on process measures, which in turn affect outcome measures. This model implies that the structural characteristics of the places where care takes place may influence the process of care so that its quality is decreased or increased. Changes in the process of care will in turn have an effect on patient’s health. In this study, the structure includes resources and infrastructure, the process involves occupational health and safety policy and procedures and outcomes are the results of safety measures in the workplace. Donabedian’s framework is used to help the researcher in decision-making during data collection, analysis and review. For example, when the researcher developed the interview tool in the focus group discussions, Donabedian framework approach, namely structure, process and outcome, was used.

## The objective of the study

This study aimed to explore and describe perceptions of nurses on occupational health hazards and safety practices in Ditsobotla public hospitals of North West province.

## Research methods and design

An exploratory, descriptive, qualitative research design was undertaken in this study. An explorative design allowed the researcher to identify key issues regarding nurses’ perceptions on occupational health hazards and safety practices using Donabedian structure, process and outcome. Qualitative exploratory design was used to gain insight, to explore the richness and complexity and helped to shed more light on the various ways in which the phenomenon manifests (Burns & Groove 2013:23). The descriptive design was used to describe the ‘who, what and where’ aspects of events (the perceptions of nurses on occupational hazards and safety practices).

### Research setting

This study took place in Ditsobotla, which is a subdistrict of Ngaka Modira Molema District in General De La Rey hospital (GDLR) and Thusong hospitals. The researcher chose these public hospitals because of their accessibility and they were the only district hospitals situated in the deep rural area where farming is the main economic activity. These hospitals are estimated to serve a population of 149 737 (Department of Health 2015:19). The package of services provided in these two district hospitals includes trauma and emergency care, inpatient and outpatient visits, paediatric and obstetric care. Staffing includes advanced midwives, midwives, enrolled nurses and nursing auxiliaries, social workers, a dietician, full-time medical officers and visiting specialist obstetricians (Department of Health 2015:19). General De La Rey hospital and Thusong hospital open 24 h a day, 7 days a week and the services are free of charge. All psychiatric cases are transferred to Bophelong psychiatric hospital.

### Population and sampling

The total population of nurses working in Ditsobotla public hospitals (GDLR/Thusong) consists of 150 nurses of all categories (registered nurses, enrolled nurses and enrolled nursing auxiliary), 90 nurses from GDLR and 60 from Thusong hospital. Nurses were the targeted population in this study because occupational hazards and risks are common in the nursing profession and nurses are the only healthcare workers who spend 24 h with patients. In this study, the researcher used non-probability sampling called purposive, where the researcher selected different categories of nurses based on her own judgement to participate in the study. The two selected public hospitals in Ditsobotla subdistrict were chosen purposively because they were the only existing district hospitals in this area. A minimum of 15 participants who met the inclusion criteria were included in the study (see Table 1), 7 participants were from Thusong hospital and 8 were from GDLR. Brink, Van der Walt and Van Rensburg (2018:144) found that 15 participants from a homogenous group were sufficient to reach data saturation. The researcher grouped the participants based on their unit or area of work. Four focus group discussions were conducted, the first group consisted of three participants and the last three groups consisted of four participants of similar categories working in the same unit or area. The sample size was determined by data saturation, which was reached after three focus group discussions, with one more additional focus group discussion to confirm the point of saturation.

**TABLE 1:** Demographic data.

Demographic factors	Number	Percentage
**Age**		
20–29 years	9	60.0
30–39 years	3	20.0
40–49 years	2	13.3
50–59 years	1	6.7
**Gender**		
Male	3	20.0
Female	12	80.0
**Race**		
Black people	13	86.7
White people	2	13.3
**Occupational rank**		
Registered nurses (RN)	8	53.3
Enrolled nurses (EN)	3	20.0
Enrolled nursing auxiliary (ENA)	4	26.7
**Unit of work**		
Paediatric	2	13.3
OPD/casualty	2	13.3
Medical/surgical	7	46.7
Maternity	4	26.7
**Years of experience**		
From 2 to 10 years	12	80.0
From 11 to 20 years	1	6.7
Above 20 years	2	13.3

OPD, outpatient department.

### Inclusion and exclusion criteria

In this study, the inclusion criteria were Registered Nurses, Enrolled Nurses and Enrolled Nursing Auxiliary’s, who had two years of working experience in Ditsobotla public hospitals, registered with South African Nursing Council and willing to participate in the focus group discussion. During recruitment process, the researcher used gatekeepers and mediators. Gatekeepers were the Chief Executive Officer (CEO) and the Nursing Service Manager of the hospitals, and mediators were administration clerks. The gatekeepers helped the researcher to identify two mediators willing to assist with the recruitment process. The researcher had a meeting with the gatekeepers and mediators to inform them about the study. The researcher explained the expected role of the mediators in the study and trained them in the recruitment process. Therefore, mediators were asked to sign the confidentiality agreement and the researcher gave them the information sheet and recruitment flyers for the study. The mediators’ role was to advertise the study to all nurses in Ditsobotla public hospitals by distributing the flyers during staff meetings and posting them on the notice boards of the hospitals. The researcher emphasised to the mediators, who had social contact with participants that their role was to simply disseminate recruitment flyers, not to pressure or coerce potential participants into joining the study. Participants who were interested in participating in the study were asked to contact the researcher via the contact details provided in the recruitment flyer. Once the potential participants who met the inclusion criteria were identified, the researcher set the meeting with the participants to discuss the study. The researcher presented the important information of the study and participants were given time to ask questions. Participants who were interested received an informed consent form to voluntarily sign at their convenient time and were given a week to read and returned the signed consent to the mediators. After a week, participants were contacted to set a date and time convenient for them to participate in a focus group discussion.

### Data collection

The researcher used semi-structured focus ground discussions to collect data to explore perceptions of nurses on occupational health hazards in the workplace. Data collection was performed after receiving permission from the Department of Health, Hospital management and obtaining consent from the participants to be interviewed and tape-recorded. Participants were grouped according to their work areas. During focus group discussions, participants shared their thought with one another, generated new ideas and considered a range views before answering (Brink et al. 2018:144). Focus group discussions were conducted from July to September over a period of two months in GDLR and Thusong nurse’s boardroom of North West province between 14:00 and 16:00. Four focus group discussions were conducted, the first group consisted of three participants and the last three groups consisted of four participants, thus making a total of 15 participants, and each focus group discussions lasted for 60–90 min until data saturation reached. The researcher chose a small group of three to four participants in order to be able to manage them. Researcher conducted focus group discussions using English, which was preferable language for all participants. The researcher used audio recorder to capture data, and field notes were also taken to capture information regarding non-verbal behaviour, such as gestures between group members, eye contact, posture and fidgeting during discussions and integrated with the data obtained from the group discussion. Permission to use the recorder was obtained from the participants. At the end of interviews, the participants were thanked.

### Data analysis

Qualitative data analysis is an on-going process, which continues throughout data collection. The researcher analysed and discussed data as one set of data using the framework proposed by Creswell and Clark (2017:140).

The researcher familiarised herself beforehand with the data by transcribing the audio recordings. During the analysis, the researcher immersed herself in the data, by reading and re-reading the transcripts:

- Each transcript gave the researcher an overall picture and explored the underlying meaning.
- The researcher read the transcripts and highlighted features relevant to the research questions. During the reading, the researcher identified common patterns and themes amongst the data. The researcher achieved this by highlighting the recurring themes, language and opinion of the participants.
- Thereafter, the data were grouped and codes were provided for each group.
- The researcher then searched for themes from meaningful patterns (by linking codes) that emerged from the data and relevant to the research question.
- Codes were given to topics and subtopics and added along the appropriate segment in the text.
- The most descriptive wording was selected for topics and converted into categories.
- Corresponding data were grouped under each category to highlight themes and subthemes.

Three categories were identified as follows: Perceptions of nurses’ occupational hazards in the health settings, Donabedian framework on assessing the quality of care in relation to occupational hazards and occupational health and safety (OHS) practices. Eight themes and subthemes were emerged (see Table 2).

**TABLE 2:** Nurses’ perceptions on occupational hazards and safety practices in the workplace.

Category	Themes
1. Perceptions of nurses occupational health hazards in the health settings	1.1 Physical hazards 1.2 Biological hazards 1.3 Psychosocial hazards 1.4 Mechanical hazards
	2. Donabedian framework on assessing quality of care in relation to occupational health hazards	2.1 Donabedian structural model 2.2 Donabedian process model 2.3 Donabedian outcome model
	3. Occupational Health and Safety practices (OHS)	3.1 Compliance and non-compliance of Occupational Health and Safety policy

#### Respect for participants

Individuals are autonomous; in other words, they had the right to self-determination. This implies that an individual has the right to decide whether to participate in a study without the risk of penalty (Brink et al. 2018:29). In this study, the researcher ensured this by treating participants with respect and ensured the maintenance of their safety and well-being at all times. Participants were informed that only the researcher would conduct focus group discussions and would use an audio recorder.

##### Principle of beneficence

According to Brink et al. (2018:29), to adhere to this principle, the researchers needed to secure the well-being of the participant, who had a right to protection from discomfort and harm. In this study, participants were protected from physical or psychological harm at all the time. They were told that they were free to withdraw from the study at any time they felt exhausted.

##### Principle of justice

Brink et al. (2018:30) referred this principle as, ‘the fair selection and treatment of the participants during the study’. In this study, participants were fairly selected and treated equally. The researcher respected and honoured any agreement they made with participants.

## The Study’s findings

### Category one: Perceptions of nurses on occupational health hazards in the health settings

Participants revealed the following occupational hazards that were divided into four themes, namely biological hazards, physical hazards, psychosocial hazards and mechanical hazards.

#### Theme 1: Physical hazards

All participants perceived occupational health hazards as risks or dangers accepted as consequences of a particular occupation. Furthermore, they stated that occupational health hazards could be injuries, illnesses, or diseases that one is exposed to whilst on duty and can result in physical injuries. Some participants highlighted the following:

‘When general workers are cleaning the floor the floor is wet, they do not put the sign and someone can pass through the wet floor, which is slippery, thus leading to physical injury.’ (Participants E, female, medical ward)

‘I was working in night shift when I experienced flood in the hospital and nurse could not move around and this affected the routine and I was afraid that patients can slip and fall.’ (Participant B, female, medical ward)

‘I was working in night shift with diabetic patients monitoring their blood glucose and because of nurse’s negligence, needle was left inside the glucose machine and I mistakenly pricked myself with that needle.’ (Participants A, female, surgical ward)

Participants understood what occupational injuries are and factors leading to health hazards in the workplace. The results show that participants are exposed to risk of slips, fall and needle stick injuries in their working areas, and they carry out their work in high-risk environment even if they observe the precautionary measures, they are vulnerable to accidents and hazards.

#### Theme 2: Biological hazards

Biological hazards are a very serious concern in the nursing profession not only in GDLR and Thusong hospitals but any institutions in the world. Most of the participants during focus group discussions stated that they are exposed to infections such as hepatitis B and C, HIV and AIDS and COVID-19 as public health concern worldwide. The sentiment of participants is as follows:

‘As a result of shortage of sharps containers, nurses walk with the needle from one area to another and this can result in sharps or needle injury, which can cause risk of HIV and hepatitis infection.’ (Participant J, male, antenatal care and post-natal care)

‘I am exposed to COVID-19 at work because I am working with different people, not only patients but also the mixture of other healthcare workers, if I come in contact with COVID-19 patient I didn’t go home for 7 days. I stayed at nurses’ home because I am afraid of transmitting the virus to my family.’ (Participant G, female, surgical ward)

‘Working in an environment that is highly contagious such as TB and HIV area, places one at risk of contracting infection and puts families at risk as well.’ (Participant E, female, medical ward)

Evidence shows that participants were exposed to dangerous viruses including the worldwide pandemic called COVID-19. Nurses perform their duties of organising the environment, coordinating the work and providing direct care for patients with highly infectious diseases, thereby ensuring tasks such as good hygiene and administering medication. All of these activities put them at risk of contracting infectious diseases.

#### Theme 3: Psychosocial hazards

The work environment has psychosocial hazards, which include workplace violence and stress. Participants expressed that they are entitled to work in a safe and healthy environment. The following are examples of sentiments verbalised by most participants:

‘Sometimes we admit pregnant women with psychiatric disorder in our unit and during the middle night they threaten us by violent acts and without male nurse, we end up calling the security to assist us in sedating patient.’ (Participant J, male, antenatal care and post-natal care)

‘I have once witnessed a nurse in a psychiatric ward being assaulted by a patient and that nurse ended up in resigning from the organisation.’ (Participant K, female, medical ward)

‘Stress in our area is caused by working hours, nurses work 200 hours per month and even 210 hours per month without any form of compensation’ like any other healthcare workers.’ (Participant D, female, medical ward)

The environmental risk factors associated with assault of nurses are inadequate training, staffing patterns, time of day and containment practices. Most of the female participants reported to be the victims of verbal abuse more often than male nurses. Nursing by its nature is one of the most stressful professions owing to the emotional nature of patient demands, long working hours and inter-professional and inter-personal conflict.

#### Theme 4: Mechanical hazards

Musculoskeletal injury includes sprain, strain or any kind of injuries to bones, tendons, muscles, ligaments, soft tissue and nerves. Nurses reported back injury and back pain as the most common concern as they affect negatively the quality of life and productivity of the organisation. Many participants had personal experiences of such injuries. This was how participants expressed it:

‘I was working night duty where I experience once experienced slip at work, this happen as a result of floods in the whole hospital, wards when I slipped trying to move patients and nurse could not move around and these affect the routine and from one of nurse slip and fall when trying of move patients.’ ‘ward to another.’ (Participants L, female, gynaecological ward)

‘Previously nurses who used to be the porters performed the duties of porters in the hospital, many nurses were complaining of back pain or injuries because of heavy workload.’ (Participant K, female, antenatal and postnatal care)

‘Shortage of resources such as cord bed in our hospital resulted in risk of falling for epileptic patients.’ (Participant J, female, antenatal and postnatal care)

Amongst other professions, nurses reported to be at risk of mechanical injury. Participants indicated that the most common cause of this injury is because manual handling, which requires force to pull, push or lift patients. Nurses from the specialised area are prone to these hazards because of the nature of their work and other reason is shortage of staff, which impose a large amount of burden to nurses.

### Category two: Donabedian framework on assessing the quality of care in relation to occupational hazards

The following themes were identified: Nurses’ perception regarding the Donabedian structure, process and the outcome model.

#### Theme 1: Donabedian structural model

Structure in the Donabedian model refers to the factors that influence the context, which in this study were prerequisites such as hospital infrastructure and resources (Donabedian 1988:1744). Majority of the nurses indicated that the arrangements in the patient, nurse and medicine preparation rooms were insufficient. Poor maintenance of infrastructure was also found to be a factor affecting their health. The nurses further stated that they were exposed to various work-related accidents and risks as a result of having to work in narrow patient rooms:

‘The infrastructures are going beyond replacement, because the buildings are too old and ceilings are falling down, paints are peeling off, walls are gapping, there is not enough light and this can contribute to occupational hazard.’ (Participant C, male, medical/surgical ward)

‘We are sharing bathroom with the patients and their relatives because of poor infrastructure and this demotivates us at work because it can lead to risk of infection.’ (Participant A, female, medical surgical ward)

‘Our hospital does not have isolation area, so we end up mixing together patients with infectious and non-infectious diseases.’ (Participant O, male, outpatient department/causality)

Nurses reported that they were not comfortable in their working area because of poor infrastructures. It is indicated that old infrastructure affects their health as they were exposed to various work-related risks as the structure of wards is too small and this can lead to the risk of infections as a result of working with many patients in a small room.

#### Theme 2: Donabedian process model

According to Donabedian (1988:1745), process refers to that which is being performed. Process describes how structure is put into practice. In this study, the process was the resources that are put in place including staff and personal protective equipment (PPE). Participants highlighted the shortage of staff as a serious concern in health facilities and sometimes they work without protective equipment and they improvise in order to provide patient care. Participants verbalised similar comments as follows:

‘I was working in an emergency unit, where I had to conduct the delivery without apron, I had to improvise in order to continue with my duty and I ended up wearing patient gown in order to protect myself from blood-borne infections.’ (Participant A, female, medical surgical ward)

‘Infection prevention and control requires us to wash our hand before and after any surgical procedure, sometimes this is difficult in my unit because we do not have hand soap and warm water but in order to minimise infection we use the scrub that is available by that time.’ (Participants E, female, medical ward)

‘I consider the shortage of personal protective equipment for healthcare workers as an occupational hazard, because if there is no proper utilisation of gloves, plastic apron and eye goggles; I am prone to various cross infectious diseases such as tuberculosis, COVID-19 and meningitis.’ (Participant G, female, surgical ward)

‘I feel that shortage of staff is because of unfilled vacant because staffs are resigning and some absent themselves from work because of personal reasons.’ (Participant O, male, outpatient department/causality)

Nurses’ perceived shortage of staff is an obstacle for the provision of quality patient care and undermining the goals of the health system globally. Nurses also indicated that working without PPE pose them at risk of infectious diseases and some had to take prophylaxis.

#### Theme 3: Donabedian outcome model

An outcome is the desired or undesired change that occurred because of the results of process (Ghaffari et al. 2014:50). In this study, outcome was the results of the process in place. Nurses absent themselves from work because of the shortage of staff in the units. Most staffs do not report on duty because they are dissatisfied at work because of many reasons. Comments highlighting these problems were verbalised by most participants:

‘In my unit, people report sick almost every month because of working environment that is not conducive.’ (Participant Q, female, labour ward)

‘Majority of people are resigning and as a result we are pulling very hard to be able to render the best quality and holistic care to patients.’ (Participant D, female, medical ward)

‘We are also losing most of the qualified professionals such as specialised nurses and doctors because of shortage of resources.’ (Participant Q, female, labour ward)

Shortage of nurses results in heavy workloads, excessive mandatory overtime, unsatisfactory physical state of hospitals and demands by management, authorities, patients and visitors made it almost impossible for nurses to function effectively, prompting them to leave their employer. Absenteeism may be directly related to work conditions, reflecting on quality and productivity and on the personal life of the nursing profession. Participants highlighted that staff are resigning because of various problems in the workplace, for example, nurses moved from developing areas to developed areas in order to find better opportunities for themselves.

### Category three: Occupational health and safety practices

This category consists of one theme: compliance and non-compliance of nurses on OHS policy and two subthemes knowledge and practices and education and training on OHS were highlighted.

#### Theme 1: Compliance and non-compliance of nurses on occupational health and safety policy

Participants highlighted that compliance on the policies and guidelines of OHS lies on the personnel designated to do the job. Although each of us is obliged to adhere to the implementation rules and regulation towards infection control, but implementation of such guidelines is the best shown when people are designated to do their task. Comments similar to this was verbalised by most participants:

‘I feel that occupational health and safety, quality assurance and infection prevention and control team are not training us enough with those policy and procedures in the workplace.’ (Participant M, female, gynaecological/maternity ward)

‘There are rules and regulation for COVID-19 that are put in place, however, I feel that they are not dynamic or stable because they change now and then. So it becomes difficult sometimes to implement and comply with those policy and guidelines.’ (Participant B, female, medical/surgical ward)

‘There is a lack of in-service training on the waste control and prevention management.’ (Participant J, female, antenatal care and postnatal care)

‘Training programme is needed for new employees when new equipment or processes are introduced and procedures have been revised and updated. For example, managers must design a training programme for new employees in a workplace in cooperation with the safety measures in the facility.’ (Participant B, female, medical/surgical ward)

Nurses perceived that the employer in collaboration with occupational health and safety team should arrange training for all employees in order to provide them with knowledge on health and safety matters as there is lack of training in the organisation. It is important to educate and train newly employed staffs on the dolling and doffing of personal protective equipment in order to become familiar to practising the correct measure of infection prevention and control. Some nurses indicated that they adhere to universal precautions including COVID-19 regulations such as wearing face mask, sanitising and hand wash procedures is difficult because of shortage of resources.

## Discussion of the findings

In this study, the findings are discussed as follows: category 1: Perceptions of nurses on occupational health hazards in health settings and category 2: Donabedian structure, process and outcome model and lastly occupational health and safety practice in the health settings.

### Perceptions of nurses on occupational health hazards in the health settings

The objective of this study was to explore and describe perceptions of nurses on occupational health hazards in the health settings. The study revealed that nurses are exposed to occupational diseases, which are illnesses developed from exposure to biological hazards such as bacterial infections that causes tetanus, tuberculosis, gonorrhoea and virus (hepatitis, HIV, COVID-19), physical and mechanical hazards risks such as sharp injuries, fall, cuts, abrasions and muscular and skeletal disorders (MSDs) and psychosocial factors that are present in the work environment or are encountered in the course of their employment.

These finding are largely comparable to previous studies conducted in low- and middle-income countries such as Uganda, which reported that sharp-related injuries and stress were the major health-related hazards experienced by HCWs (Adib-Hajbaghery & Lofti 2013:75; Ziraba et al. 2010:13). Rapiti, Pruss-Ustun and Hutin (2014:24) stated that sharp injuries to HCWs increase the risk of hepatitis B and C and HIV infections amongst HCWs. High incidence of infectious diseases was reported in developing areas as a result of challenges to implement OHS practices because of scarcity of resources (d’Ettorre & Pellicani 2017:342). Therefore, the need to implement safety practices in the workplace is necessary.

Participants also experienced stress in the workplace because of long shifts, they work 12 h/day and sometimes going without lunch as a result of heavy workload. This is consistent with the findings by Edward et al. (2016:299) who stated that prolonged exposure to environmental and situational hazards resulting in work-related stress contributes to emotional exhaustion, depersonalisation and a lack of personal accomplishment. The workplace should be designed in such a manner to prevent invasion, harassment and stress free against nursing professionals.

### Donabedian framework on assessing the quality of care in relation to occupational hazards

This study revealed predictors of occupational health hazards based on Donabedian structure, process and outcome. The majority of the participants indicated poor infrastructure and resources as a major problem in the health settings. Structural problems may result in nurses leaving their jobs. As stated in this study, it was found that nurses suffered serious injuries and accidents in poor work environments. This is especially the case when the physical areas are not spacious enough to provide freedom of movement, a situation that could lead to needle injuries or physical risks. In a study conducted by Nevhutalu (2016:138), patient and staff confirmed that some departments had an unacceptable physical environment (e.g. dirty toilets for delivery of quality healthcare).

Personal protective equipment is defined as the most important aspect of protection against professional risks; however, it has been observed that employees are neither properly nor sufficiently provided with PPE (Kang et al. 2017:17). A study conducted by Mokoena in one of the public hospitals of Limpopo province revealed that the lack of material resources, equipment and supplies (e.g. glucometers for monitoring blood glucose and needles for lumbar puncture and investigating or diagnosing meningitis), resulted in prolonged patients stay in the hospital as patients were referred to other hospitals for investigating delayed diagnosis and treatment (Mokoena 2017:67). Lack of resources should be the hospital executives’ responsibility, providing a supportive environment for nurses, not nurses’ problem to handle (Dutra, Cimiotti & De Brito Guirardello 2018:68).

Staff shortage in some departments has put job pressure and inability to engage new staff on proper job orientation and training before commencement of their duty. Study by Kumari and De Alwis (2015:75) revealed that staff issues in particular staff shortage result in higher workloads, which affect job satisfaction. This is relevant for developing contexts such as South Africa, where staff shortage evident in the large number of vacant nursing posts (Makola, Mashegoane & Debusho 2015:30) could explain the stronger relationships between work stress and job satisfaction.

### Occupational health and safety practices

The *Occupational Health and Safety Act*, Act 85 of 1993, requires the employer to provide and maintain as far as reasonable and practical work environment that is safe and without risk to the health of employees. Hospital responsibilities, such as risk assessment, health screening and monitoring and informing employees are supposed to be carried out by the ‘Occupational Health and Safety Team’ in accordance with the law (Gazette Official 2012:8). From the nurses’ statements, it was understood that the ‘Work Health and Safety Board’ operating under the Employee Health and Safety Unit of the hospital do not fulfil their obligations.

According to Asa, Marcus and Jones (2013:13), a safety culture reflects individual, group and organisational attitudes, values and behaviours concerning safety. Safety management relates to the formal safety practices and responsibilities documented in a safety management system (Asa et al. 2013:13). Nurses stated that compliance on safety culture was the fault of the supervisor for not training staff on infection prevention and safety precautions in the health settings. A well-developed safety culture in an organisation enables the maintenance and improvement of safety performance, with an emphasis on safety work and improvement processes for safety.

This perception implies that knowledge pertaining to OHS is important and can play a role when employers want to prevent occupational injuries. The implication is that if employees receive education related to occupational health and safety issues they will know what to do to prevent injuries in the workplace. However, there should be administrative guidance and training on how healthcare professionals can deal with such hazards in the workplace. This is consistent with the finding by Butler (2020:5) that the best places to work are places where nurses are provided with training and opportunities to develop. Nurses require a healthy working environment, and emphasis should be placed on maintaining their health, rather than only expecting them to be productive.

### Recommendations

In the light of the findings, the following were recommended by the researcher:

- Each organisation should have a comprehensive training programme for nurses to update their knowledge on various occupational hazards faced and their role in minimising these risks for practicing safe nursing.
- Nurses should be provided with in-service training regarding OHS policy and adequate person protective equipment’s should be available at all time.
- Further studies should be undertaken to analyse the after-effect of work-related injuries and illnesses on the nurse.

### Limitations of the study

This study was constraint on nurses’ perceptions on occupational hazards. A follow-up study on perceptions regarding occupational hazards should incorporate all HCWs in the hospitals. The qualitative data were limited to the information that the participants had provided in the focus group discussion. As a result of the fact that this study was conducted in level 1 district hospitals of North West province of South Africa, transferability to larger populations cannot be made from this study.

## Conclusion

This study serves to provide information on the perception of nurses on occupational health hazards and safety practices in the workplace. Nurses perceived different occupational hazards in the workplace and highlighted that they are regularly exposed to various hazards because of a shortage of resources in their working units. In referring to the findings of this study, it is recommended that nurses should be trained on safety measures to protect themselves against occupational health hazards.
